# Supplementary material for: Investigation of genetic variants and causal biomarkers associated with brain aging
Source: Sci Rep. 2023 Jan 27;13:1526. doi: 10.1038/s41598-023-27903-x (PMC9883521; doi:10.1038/s41598-023-27903-x)
Supplement: Supplementary file 1 — Supplementary Information. [file 41598_2023_27903_MOESM1_ESM.zip › Supplementary_Materials.pdf]

Investigation of Genetic Variants and Causal Biomarkers Associated with Brain Aging

Jangho Kim, Junhyeong Lee, Kisung Nam, Seunggeun Lee*

Graduate School of Data Science, Seoul National University, Republic of Korea

Supplementary Materials

(a) SAIGE result on the fornix volume (single-variant test with array-genotyped and imputed data)

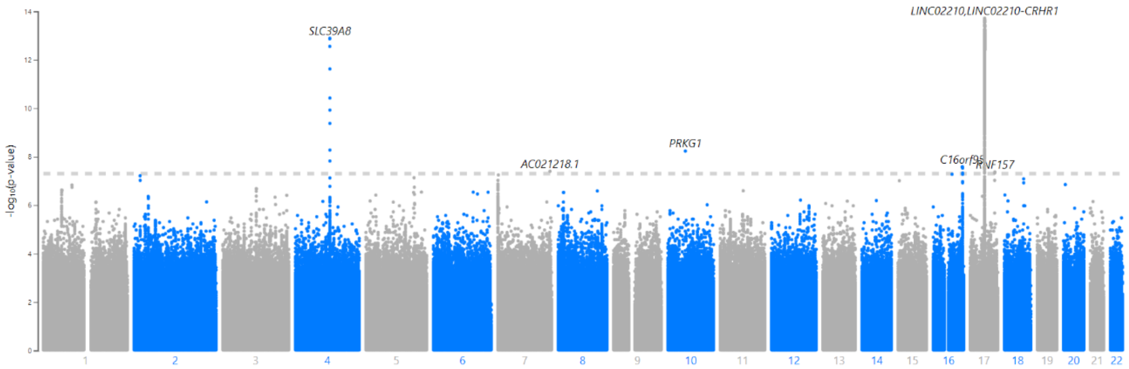

(b) SAIGE result on volume in the lower part of the thalamus (single-variant test with array-genotyped and imputed data)

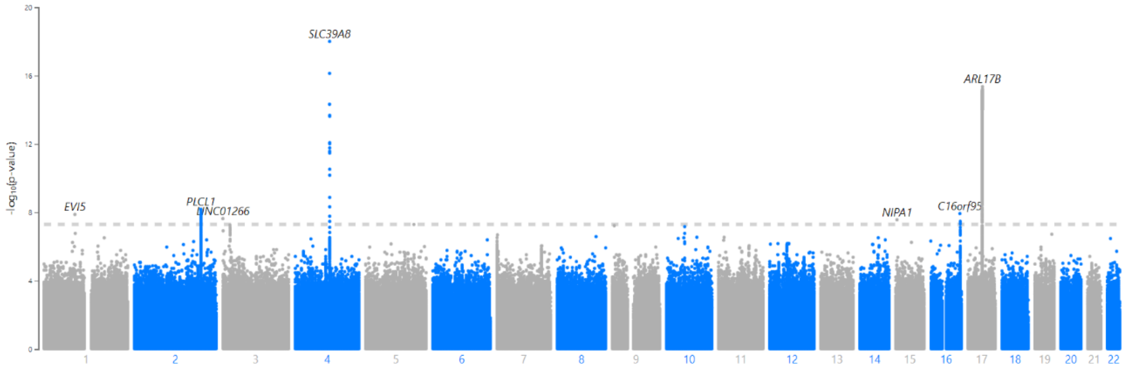

Supplementary Figure 1 GWAS results on the average voxel values in the fornix and the lower part of the thalamus.

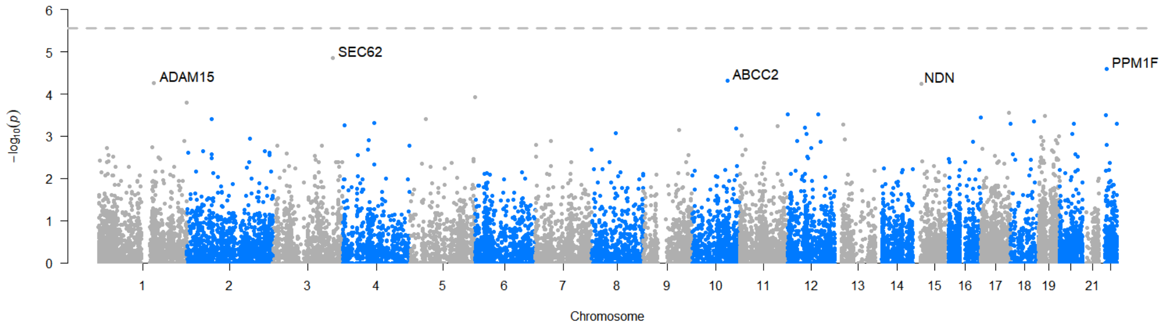

Supplementary Figure 2 Manhattan plot of gene-based tests with the WES data on the delta age.

(a) Inverse variance weighting method

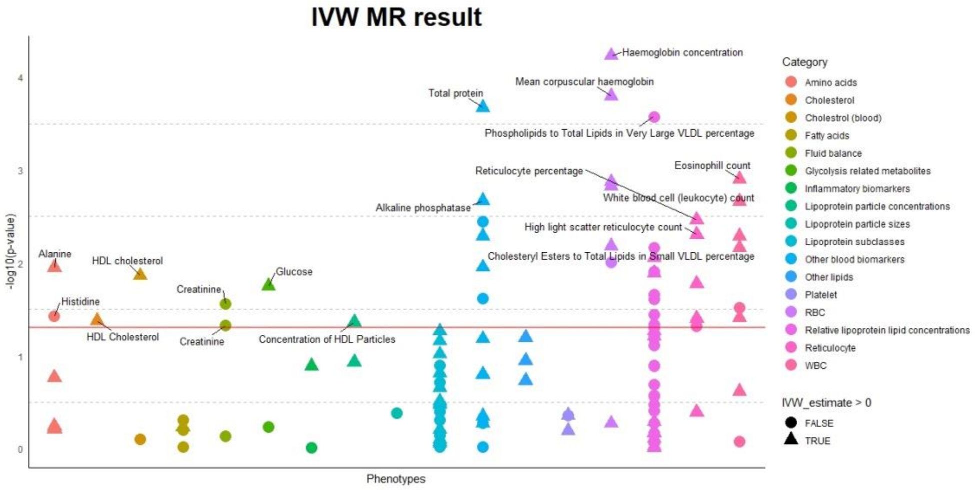

(b) Weighted median method

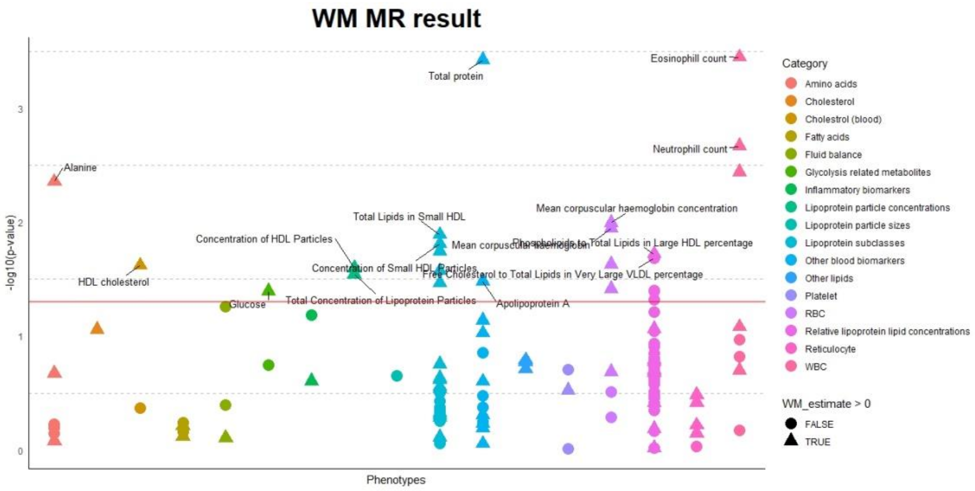

Supplementary Figure 3 PheWAS plots of the linear MR causal estimates from (a) Inverse variance weighting method and (b) Weighted median method

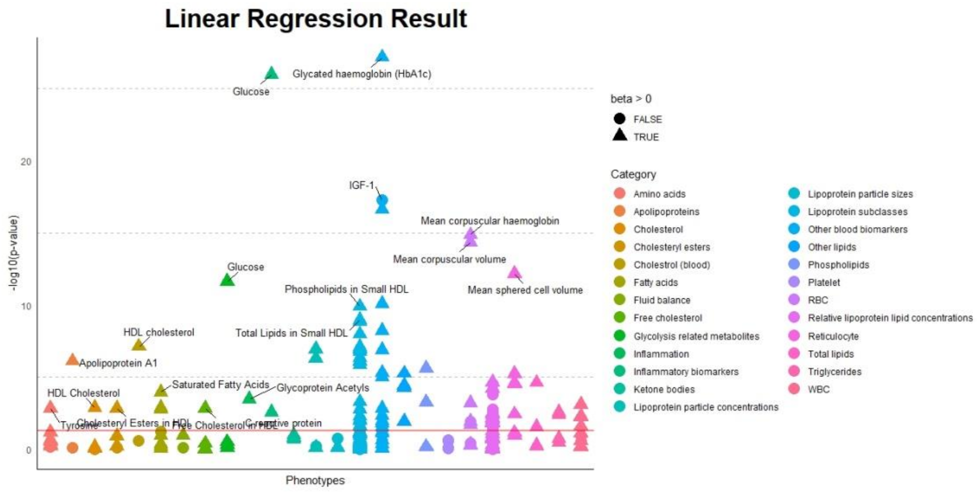

Supplementary Figure 4 A plot of p-values of each blood-chemistry and metabolomics biomarkers from the association analysis

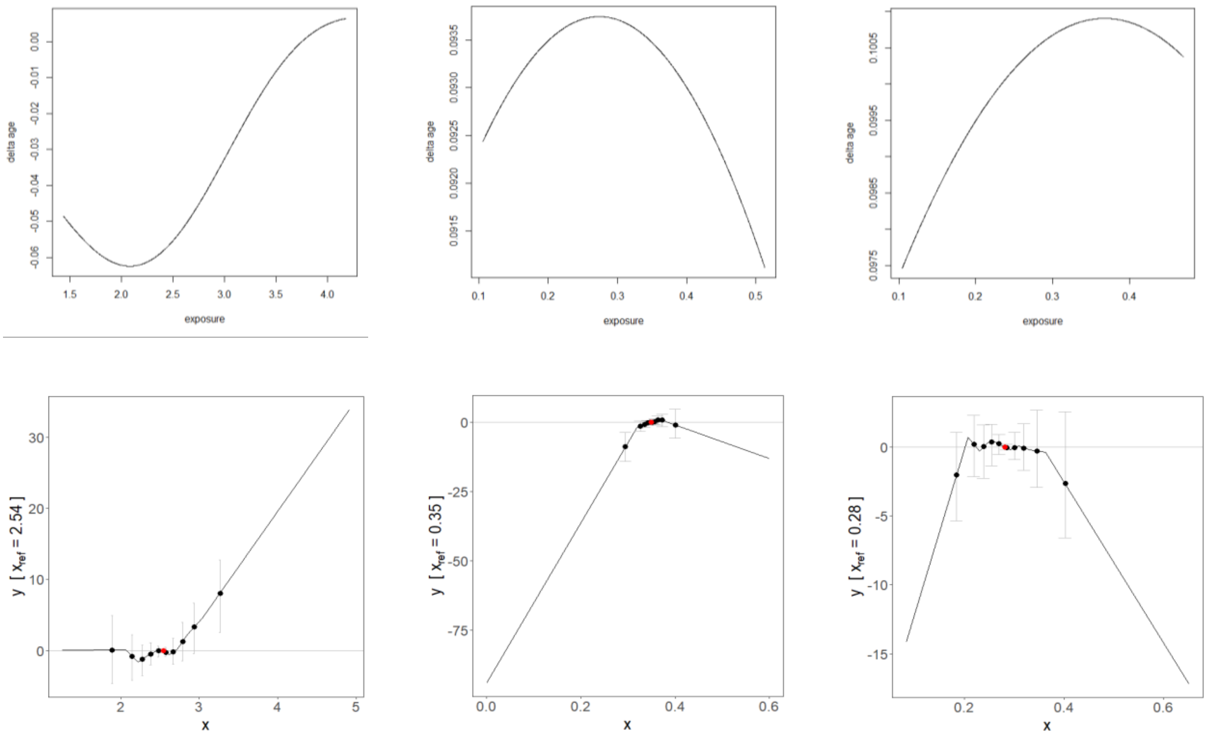

Supplementary Figure 5 Plots of the nonlinear causal relationship between the biomarkers and delta age. The first row shows the plots with kernel IV regression, and the next row shows the plots with piecewise MR. The order of the plots is total cholines, total lipids in small LDL, and cholesteryl esters to total lipids in very large HDL percentage.

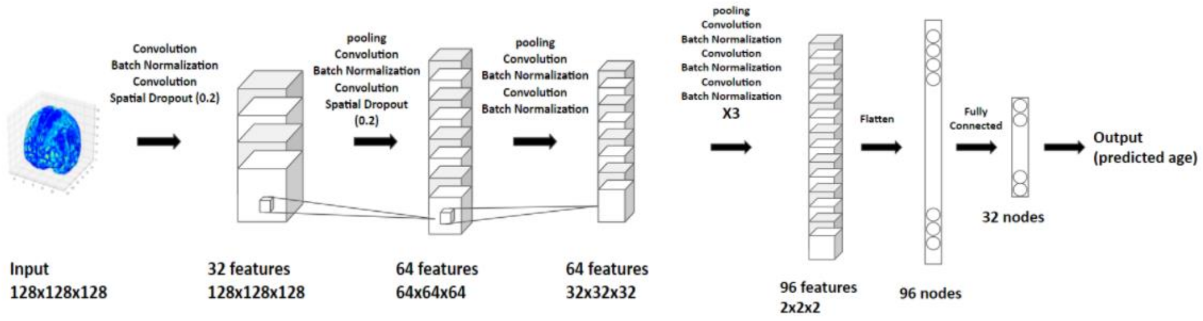

Supplementary Figure 6 The convolutional neural network structure of the age prediction model

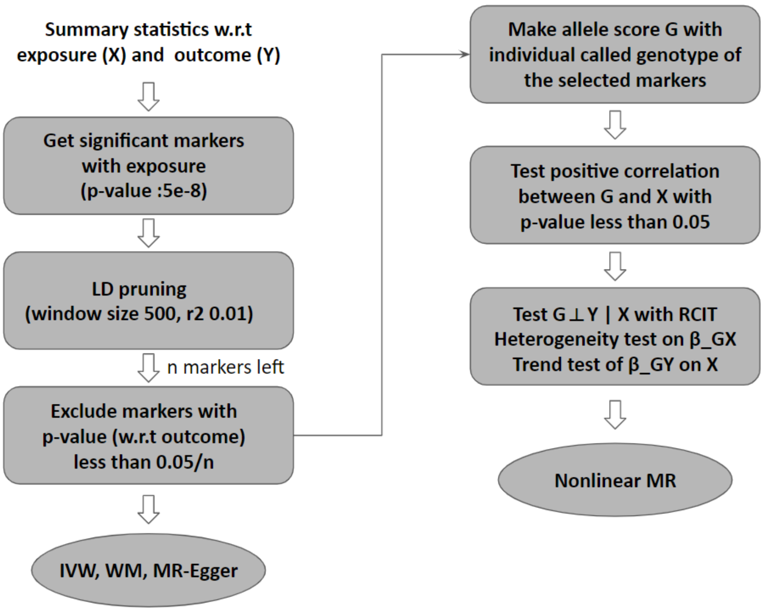

Supplementary Figure 7 Workflow of the linear and nonlinear MR analysis
